# Hyperparameter selection for dataset‐constrained semantic segmentation: Practical machine learning optimization

**DOI:** 10.1002/acm2.14542

**Published:** 2024-10-10

**Authors:** Chris Boyd, Gregory C. Brown, Timothy J. Kleinig, Wolfgang Mayer, Joseph Dawson, Mark Jenkinson, Eva Bezak

**Affiliations:** ^1^ Allied Health and Human Performance University of South Australia Adelaide Australia; ^2^ Medical Physics and Radiation Safety South Australia Medical Imaging Adelaide Australia; ^3^ Department of Neurology Royal Adelaide Hospital Adelaide Australia; ^4^ Adelaide Medical School The University of Adelaide Adelaide Australia; ^5^ Discipline of Surgery University of Adelaide Adelaide Australia; ^6^ Department of Vascular and Endovascular Surgery Royal Adelaide Hospital Adelaide Australia; ^7^ Industrial AI Research Centre, UniSA STEM University of South Australia Adelaide Australia; ^8^ Australian Institute for Machine Learning (AIML), School of Computer and Mathematical Sciences University of Adelaide Adelaide Australia; ^9^ South Australian Health and Medical Research Institute (SAHMRI) Adelaide Australia; ^10^ Wellcome Trust Centre for Integrative Neuroimaging (WIN) Nuffield Department of Clinical Neurosciences (FMRIB) University of Oxford Oxford UK; ^11^ Department of Physics University of Adelaide Adelaide Australia

**Keywords:** applied AI, computer vision, hyperparameters, machine learning, segmentation, sensitivity analysis

## Abstract

**Purpose/aim:**

This paper provides a pedagogical example for systematic machine learning optimization in small dataset image segmentation, emphasizing hyperparameter selections. A simple process is presented for medical physicists to examine hyperparameter optimization. This is also applied to a case‐study, demonstrating the benefit of the method.

**Materials and methods:**

An unrestricted public Computed Tomography (CT) dataset, with binary organ segmentation, was used to develop a multiclass segmentation model. To start the optimization process, a preliminary manual search of hyperparameters was conducted and from there a grid search identified the most influential result metrics. A total of 658 different models were trained in 2100 h, using 13 160 effective patients. The quantity of results was analyzed using random forest regression, identifying relative hyperparameter impact.

**Results:**

Metric implied segmentation quality (accuracy 96.8%, precision 95.1%) and visual inspection were found to be mismatched. In this work batch normalization was most important, but performance varied with hyperparameters and metrics selected. Targeted grid‐search optimization and random forest analysis of relative hyperparameter importance, was an easily implementable sensitivity analysis approach.

**Conclusion:**

The proposed optimization method gives a systematic and quantitative approach to something intuitively understood, that hyperparameters change model performance. Even just grid search optimization with random forest analysis presented here can be informative within hardware and data quality/availability limitations, adding confidence to model validity and minimize decision‐making risks. By providing a guided methodology, this work helps medical physicists to improve their model optimization, irrespective of specific challenges posed by datasets and model design.

## 1 INTRODUCTION

In many fields, semantic segmentation, that is, the categorization of individual pixels within an image, is a useful but laborious task.^1^ The recent emergence of advanced Artificial Intelligence (AI) segmentation methods using Machine Learning (ML), and Deep Learning (DL), has presented new opportunities to automate elements of this work. This automation may free specialist staff time to undertake other tasks, whilst also improving accuracy and even reducing inter‐ and intra‐observer variability. ML semantic segmentation has shown utility in a wide range of fields, including transport, traffic, manufacturing, and multiple aspects of medicine such as diagnosis, treatment planning, and risk categorization.^2^

The role of the medical physicists in AI development, has been examined in depth by a recent Medical Physics special issue.^3^ In particular, the introductory paper of this issue provided a step‐by‐step machine learning guide for medical physicists.^4^ The paper defines terms commonly used in the field, identifies often encountered issues (such as the bias‐variance trade‐off and vanishing gradient problem), and finally provides both the theory and supporting mathematics of clinically useful model architectures. Another special issue article provided numerous references illustrating the potential of AI models in medical physics^5^ and detailed current conceptual “big‐picture” issues impacting the clinical adoption of AI such as data integrity, medical ethics, quality assurance, and regulatory approval and then provided a training framework to equip medical physicists to operate effectively in this space. In the same issue, a detailed guide is provided on the pros and cons of each tool, including a step‐by‐step guide for the complete process from the collection and curation of data, to the problems of applying conventional statistics to AI solutions.^6^

Using only this focus issue and easily‐obtainable online resources, any medical physicist with basic programming knowledge could create functioning ML tools. Such tools could appear to achieve the desired task and may even be implemented to support clinical functions. However, the clinical implementation of such technologies could be problematic if designers do not first understand the use cases, and even more importantly, the limitations of the model arising from choices made during model development.

### 1.1 Hyperparameters

Neural networks have become the preferred ML model architecture for semantic segmentation in medical imaging, where the values of each “neuron” are mathematical coefficients. These models require the calculation of weights and biases for multiple layers of neurons before an output layer ultimately produces outcomes. To control this calculation, several key functions are selected by the model designer including: a method to obtain training/validation/testing subsets from an initial dataset, a loss function for evaluation of outputs against a “ground truth”,^7^, ^8^ an optimization function to direct how weights are updated throughout the training process,^9^, ^10^ and an activation or transfer function to determine the activated output of each neuron or layer.^11^

The decisions made in selecting these functions are major determinants of ultimate model performance. In addition, ML designers must also choose values for a plethora of settings *within* each function. Given these function settings are not modified within an epoch of the training process, they are model “hyperparameters”,^12^ and any of these have the potential to bias the training process if chosen poorly.

Some examples of hyperparameters include: the maximum number of epochs, type of pre‐processing, as well as many others specific to a given model architecture (such as U‐net, Support Vector Machine, k‐Nearest Neighbors, and Random Forest). In the Appendix of this work, Table S1 summarizes some of the hyperparameters commonly used with the “U‐net”,^13^ a neural network architecture that is ubiquitous in current medical imaging segmentation. Given the large range of optimizers, loss functions, and other user‐defined values outlined above, an exhaustive examination of all the combinations of hyperparameter values is usually infeasible.

The comprehensive, automatic, and generalized optimization of model hyperparameters remains a field of complex and ongoing research.^14^, ^15^ Many methods are available for researchers seeking to choose suitable hyperparameter types and optimize their value.^16^ Some of these hyperparameter optimization methods include grid searches, random sampling, Bayesian optimization, evolutionary algorithms, and automated configuring networks such as nnU‐net.^17^, ^18^ Bayesian and evolutionary algorithm‐based optimization require technical and specialist knowledge to apply effectively, as well as potentially needing hundreds of thousands of runs, multiple large datasets, and/or computing power in excess of that readily available to many clinical researchers.^19^ Random sampling and grid‐search optimization are much more practically feasible for single institution models, with random sampling the preferred option as it provides approximately equivalent results, with much less training time in most cases.^17^ The major benefit to this work from using grid‐search optimization is twofold: completeness and simplicity. Grid‐search systematically and completely searches the chosen hyperparameters, ensuring the “optimal” set is found, and although random sampling more broadly explores hyperparameter space the randomly chosen combinations may not produce a fully optimized model. The simplistic nature of grid‐search optimization makes it a readily accessible approach for those new to hyperparameter optimization. A grid‐search approach is also applicable to all different types of ML models and easily scalable, so a targeted form of grid‐search optimization can be used in any model during development.

Although theoretically desirable, the technical and practical limitations associated with automated optimization of semantic segmentation models are likely to be prohibitive in many cases. In particular, the dataset requirements are likely to be prohibitive, with limited, small‐scale sets of labelled images most common when clinical staff are trying to create, optimize, and implement their own ML tools. Thanks to a recognized proficiency in programming and mathematics, clinicians may understandably look to medical physicists for assistance when seeking to develop ML models for clinical problems. With development likely subject to data and computing power limitations, there is a risk that these projects create models that appear to assist with the specific clinical problem, but also contain greater hyperparameter selection biases than those developed with a larger volume of training data. It is important, therefore, that these staff understand what options are available for hyperparameters, their appropriate use cases, and basic optimization approaches. Objective investigation of hyperparameters such as that proposed here, are likely to generate large output datasets, which may be time consuming to analyze thoroughly. To simplify this random forest regression can be used, where hyperparameter impact is analyzed against model performance metrics. Regressive analysis is a commonly used statistical process many medical physicists are already familiar with, and its application to this optimization task quickly and fairly compares each hyperparameter's impact on model performance. The presentation of these selections, concisely and transparently, is in line with the best practice recommendations for AI publication in both medical physics and broader medical imaging.^20^, ^21^

In this work, a *process* is developed for guiding medical physicists to investigate hyperparameters and evaluate the reliability and stability of a chosen hyperparameter set. The use of a relatively small dataset and simplistic machine learning approach maximizes accessibility and pedagogy for those new to the field, demonstrating simple optimization to readers through a sample use case. By presenting this process transparently with code^22^ and input images^23^ available to readers, the impact of hyperparameter selections will be demonstrated, guiding grid‐search optimization of model hyperparameters, and a starting point for further code development. Importantly, the developed process should be of benefit to most models, rather than simply highlighting specific hyperparameters of greatest influence to the case study used. Identifying specific hyperparameters as most important would only be true for a narrow set of model parameters and input data and largely irrelevant to other researchers.

**FIGURE 1:**
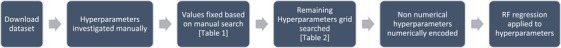
Overview of the investigation process used.

With an exhaustive hyperparameter list practically infeasible, individual experience and literature can be used to select starting values. Selecting some hyperparameter starting values and undertaking a sensitivity analysis to identify which hyperparameters seem to have little effect on training, can substantially reduce the training time required. This initial “manual search” then allows some hyperparameters to be held static throughout the rest of the process. The hyperparameters that are optimized during an initial manual search are included in Table 1, whilst the hyperparameters subject to grid search optimization are included Table 2. An overview of the development process is included in Figure 1 and further technical explanation of hyperparameters is provided in Appendix 1.

**TABLE 1:** Summary of the values of hyperparameters kept static in this work.

Sample size (patients)	Patch size (pixels)	Initial filters	Epochs	Cross validation method	U‐net layers	Layerwise dropout rate	Activation function	Kernel initialization	Kernel size
10	128	32	10	Leave one out	4	0.0	ReLU	HE normal	3 × 3

*Note*: For most datasets 5‐fold or 10‐fold cross validation is expected to be more appropriate. “Leave one out” cross validation was used here due to the small dataset size.

**TABLE 2:** Summary of the values of generic (top) and loss function specific (bottom) hyperparameters combinatorially varied in this work.

Learning rate	Dropout	Optimizer	Batch Normalization	Augmentation
0.001	0.1	Adam	TRUE	TRUE
0.005	0.3	RMSProp	FALSE	FALSE
0.01	0.5			

Loss Function	CCE/DSC ratio	Loss function	Beta values (background – bone – liver)	Class weights
Combo Loss	0.05/0.95	Recall Favored	2‐0.5‐0.5	Inverse frequency normalized
	0.20/0.80		0.5‐0.5‐0.5	0.5‐0.5‐0.5
	0.40/0.60		0.5‐2‐2	

## 2 MATERIALS AND METHODS

### 2.1 Hardware and software

All model development was undertaken using Python 3.7.11^24^ and TensorFlow 2.7.0,^25^ with all training performed with an Intel Core i7‐9700KF CPU and 11GB MSI RTX 2080 Ti GPU.

The dictionary‐based approach to grid‐searching was modelled on an existing Python library used for similar purposes, Autonomio Talos.^26^ Albumentations^27^ was used for data augmentation, where mentioned.

### 2.2 Dataset and patient demographics

In order to demonstrate the proposed method, a sample dataset was required. The specific dataset chosen here is not required for hyperparameter optimization, but does show organ segmentation, a reasonably typical task. The DICOM Computed Tomography (CT) dataset used here for both training and validation, is publicly available and openly downloadable, maximizing transparency for others looking to follow along.^28^ This dataset includes 10 male and 10 female patients, median age 57 [Range: 22–80], with liver tumors present in 75% of patients. A sample size less than 50 patients is common in single institution, early‐stage, AI development.^29^ However, given the 658 combinations of hyperparameter variations used for optimization, these patients represented an “effective dataset” of 13 160 patients.

Image and binary mask DICOM series have been systematically named and pre‐sorted into relevant subfolders and made publicly available under creative commons licensing on the website of surgical training provider IRCAD.^23^ Thirty‐seven different 512 × 512 binary DICOM mask segmentation series are provided with the downloaded dataset^28^ (qualifications and experience of the labelers are not specified), though each patient only has a subset of these segmentations included. Only four organs (bone, liver, portal vein, and body habitus) were segmented in nearly all (17/20) patients, body habitus was ignored here as it could be trivially segmented using thresholding. This gives a total of 2187 usable 2D images (“slices”), containing three “organ” classes, for further analysis.

### 2.3 Image processing

Scanner information and acquisition protocols were not provided in this dataset; given the variance in pixel size, slice thickness, and overall image appearance, multiple scanners were presumably used. Slice numbers per patient varied between 74 and 225; all slices encompassed 512 × 512 pixels and used a 16‐bit data representation. Though extensive resampling and/or pre‐processing might be able to correct for these image variations to produce a uniform dataset, the heterogeneity exhibited here was considered by us to be realistic and representative of datasets obtained from multiple clinical institutions, or even a single institution using multiple scanners, which is common when collecting images over several years.

To minimize the impact of overfitting that is common with small datasets and to increase the number of training datasets, various augmentations such as addition of noise or rotations were applied to the patient images prior to training. Although augmented, stored data may consume additional storage resources, saved datasets can alleviate memory issues in some circumstances.^30^ Four augmentations were applied (10% downscale, motion blur, gaussian blur, or 10‐degree rotation), each with a 25% use probability for a given image. Each augmentation was used with its default settings and the permutations of augmentation were limited to avoid overly noisy data that would undermine model convergence. Lastly, eight 128 × 128 image patches were sampled randomly from each 512 × 512 CT slice, subject to each sample overlapping with a target organ in the ground truth. Patches were not equally distributed across the image and may have contained any non‐zero amount of semantically segmented anatomy. As larger patches provide greater spatial information but require greater memory to process and can increase training time, the dimensions of the patches were selected through trial and error to use the largest possible size within processing hardware constraints. If training was to be performed using non‐augmented datasets, the generation of patches was performed during each training run to maximize patch variability.

### 2.4 Machine learning development

An existing single‐organ and single‐patient data loader^31^ was found which utilized the same dataset as employed here, written in Python using Tensorflow v1.0 and Keras. This code was updated to the software versions being used, before being modified to perform multiple organ segmentations, import all patients as an initial dataset, and change the validation approach. This expedited the data loading process of both input and ground truth datasets.

A simple but relatively standard two‐dimensional U‐net model was used here,^13^ as this work focuses only on the method applied. This was suitable due to the small dataset with low variability, but for other datasets or applications, models of greater complexity, increased depth, three‐dimensional “V‐nets” or any of several other U‐net modifications may be appropriate. In the case study presented here, the model design consisted of four convolution‐deconvolution layers, with a rectified linear (“ReLU”) activation function, HE‐normal initializer^32^ and a SoftMax output activation. The SoftMax activation function provided a probabilistic output for each class, since the segmentation of organs is a task with mutual exclusivity (i.e., if a pixel is one organ, it cannot be another) due to the multiclass problem presented.

**FIGURE 2:**
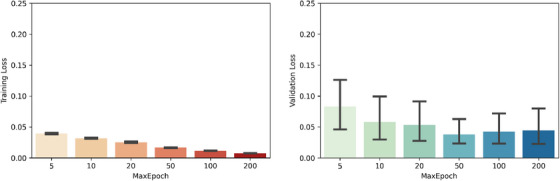
Summary of model training losses by maximum epoch number on training loss (left) and validation loss (right) loss (95% error bars shown across hyperparameter combinations manually trialed).

As testing of the hyperparameter combinations provided in Table 2 required several weeks of training, additional combinations created by varying parameters in Table 1 would then create impractically long training times. To account for this, a random search of hyperparameters was run during preliminary model training, to determine “good” values for some hyperparameters, though these values are unlikely optimal. The hyperparameters chosen for this preliminary investigation and their value ranges were chosen based on a review of similar published segmentation works,^29^ or through default argument values provided by Tensorflow. With relative model performance after hyperparameter changes being the focus of this investigation, rather than absolute model performance, one hyperparameter value was changed per training run through a narrow range that provided good results whilst maintaining practically feasible training times. To this end, model performance metrics and training times were trialed with various training set sizes of patients, and training performance was found to improve for sizes of up to 10 patients, with performance improvements diminishing beyond this.^30^ Ten epochs of training were found sufficient for stabilized model training, which can be attributed to the small dataset used, epochs beyond this provided improved model performance but at a diminishing rate. This is demonstrated by Figure 2 which shows the reduction in loss with a range of maximum epochs, a fortyfold increase in maximum epoch and training duration was seen to only reduce mean training loss from 0.04 to 0.007. Commonly used training improvement tools such as learning rate decay and early stopping were not used, as this would combinatorially increase the number of hyperparameters examined and increase training time. As this work aims to demonstrate the process, and importance, of grid‐search optimization, these hyperparameters do not add further value.

Optimizer and loss function were anticipated to be particularly influential on model performance and several options were explored during initial testing (equations for each are included in Appendix 1). The set of options investigated for the optimizer were: Adam, Adagrad,^33^, ^34^ Weighted Adam, Stochastic Gradient Descent (SGD), and Root Mean Square propagation (RMSprop).^9^ The loss functions investigated were: Recall Favored^35^ (RF), Jaccard distance,^36^ Dice loss,^36^ focal Tversky loss,^37^ and weighted Categorical Cross Entropy (CCE) with dice loss (“combo loss”).^38^ Based on these preliminary investigations, the optimizers and loss functions providing the highest metric values were Adam and RMSprop for optimizers, and combo loss and RF loss for loss functions.

The “RF loss” function is a generalized, asymmetric formalism, of the F‐score. As explained in the literature,^35^ the β parameter can be modified by the programmer to account for class imbalance by changing the weighting of precision and recall. In the case of β = 1, the RF loss simplifies to the harmonic mean of precision and recall, that is, the “F1 score” or the Dice Similarity Coefficient (DSC). If β = 0, the RF loss becomes precision. RF loss, DSC, and Jaccard distance are also mathematically similar to the Tversky index^36^ and the mathematical formulations of these loss functions is given in Appendix 1. It is important that the choices of the hyperparameters associated with these loss functions are carefully considered, since they can have a profound impact on the model's ability to train and produce accurate results. This list of loss functions should not be considered exhaustive and other options which use more diverse mathematical forms should also be considered, one such example is cross‐entropy which may provide better performance depending on model design and desired application.

Four other key training hyperparameters were investigated: dropout, batch size, batch normalization, and learning rate. Dropout is a regularization technique that stochastically disables selected neurons in a neural network to combat overfitting during training. By disabling neurons at random, the training process is prevented from relying on overly specific node configurations, so the node weights that remain are more generalized and less overfitted to the training data.^39^ Batch size is set to both suit the available computing power and potentially allow better generalization of models by avoiding overly large gradient steps during training. Though batch size may be of relevance to other applications, it was not varied as part of the sensitivity analysis here. The batch size was based on hardware limitations and was set to either 16 or 32 patches. Batch normalization normalizes layer inputs to modify the mean and standard deviation to be closer to those seen in a standardized Gaussian distribution, though ultimately the distribution remains non‐Gaussian. This step reduces variability in the inputs between layers and often improves the efficiency of model training. Finally, the learning rate scales the magnitude of changes in each step of the optimization process during training. Setting the learning rate correctly can be essential for guiding this process towards a gradient minimum that represents a generalized solution.

Leave One Out Cross‐Validation (LOO‐CV) was used for this case study as it was equivalent to the commonly used 10‐fold cross validation, producing performance metrics for all combinations of patients, and providing a more comprehensive investigation of each model hyperparameter combination. To complete LOO‐CV, each hyperparameter combination was trained on ten groups of nine patients, with the remaining patient held‐out and used for validation. Alternatives such as stratified shuffle split or k‐fold cross‐validation with other k values may be better in other applications. The primary disadvantage of LOO‐CV is that it can be computationally demanding but this was not a limiting factor for the case study here due to the small dataset used. Considering the validation technique such as discussed above, is one example of the importance of evaluating a specific use case before discarding possibly useful techniques.

The values of all hyperparameters varied combinatorially for training are summarized in Table 2, a total of 22 values across nine investigated hyperparameters, training a total of 658 combinations and 6571 folds. Training time was approximately 20 min per cross‐validation fold, with a total training time for all hyperparameter combinations of 2100 h. Once training had been completed multiple organs could be segmented from a previously unseen series of slices, from a single patient, in less than 1 min.

### 2.5 Performance evaluation

To maximize the information obtained by training and best inform decision‐making from the sensitivity analysis, collection and analysis was undertaken using as many descriptive performance statistics as possible. The loss, accuracy, categorical accuracy, DSC, Jaccard index, precision, and recall were recorded at the end of each epoch. Mean values of DSC, Jaccard index, precision, and recall were also calculated each epoch, using all three classes (including background), as well as only using the two target classes (liver and bone). These metrics were selected as they are the most commonly used metrics in the literature, based on a systematic review of clinically applied segmentation algorithms.^29^ Relevant performance values were calculated from training and validation images, with each patient used as validation when “left out” during LOO‐CV. From each of the 65 710 training epochs performed, 21 hyperparameters and 41 metric results (performance values) were obtained, producing a total of four million performance values, continually recorded throughout training steps for the purpose of debugging and retrospective analysis.

Analysis of the results presents its own challenges due to the millions of metric values generated and the need to clearly identify how each hyperparameter choice affected performance. A relative indication of each hyperparameter importance was used, calculated in comparison to all other hyperparameters, determined using a very simple code‐block to perform random forest regression analysis on metric values.^40^ After numerical encoding of non‐numerical hyperparameter values, random forest models were trained, correlating the hyperparameters used to a single performance metric. The resulting figures, shown in Figure 4, provide the relative importance of each hyperparameter on the quality of model predictions, as determined by the single performance metric. This process was repeated with varied random forest depth (5, 10, 50) and varied number of estimators (100, 1000, 5000), to produce the standard deviations shown in Figure 4. Several models of this type were constructed, each using a different performance metric output by the segmentation task. The random forest analysis investigated metric value variations and compared them between the different hyperparameters, producing a normalized output showing the chosen metrics’ sensitivity to the hyperparameter value chosen (or its’ “importance”). Though the selection of metrics is expected to be of importance to the given task, key hyperparameters are identified as those that have greater relative importance, particularly if greater relative importance is seen across multiple metrics. The mathematical interrelationship between metrics such as DSC and Jaccard Index can lead to similar relative importance, so analysis should focus on independent metrics. For this reason, developers must consider not only the relative importance of the hyperparameters, but the metric used for measurement of performance. Further, metrics have different importance and relevance to the task being undertaken, for example, segmentation of aggressive and fatal disease requires metrics that minimize false negatives, whereas other applications may be more tolerant of this in exchange for less false positives.

## 3 RESULTS

**FIGURE 3:**
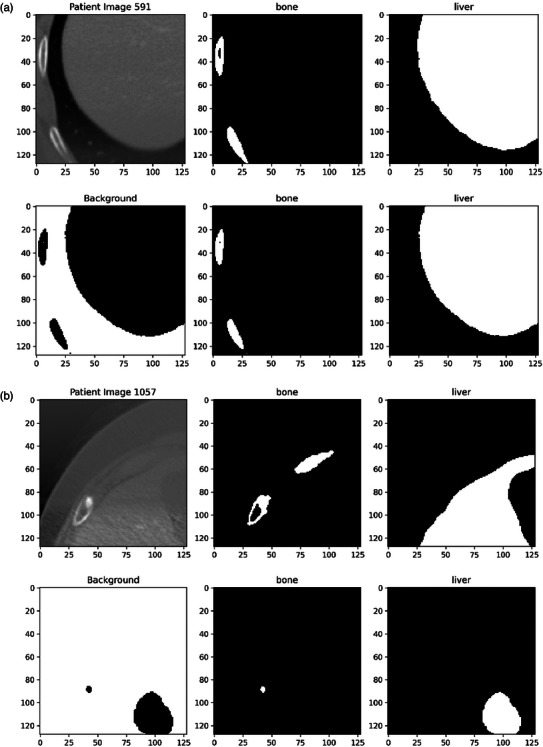
Examples of relatively successful (a) and unsuccessful (b) test set segmentations using the model from Figure 5. The top row in each contains the patient image and ground truth segmentations, with the bottom row showing model output segmentations.

Results presented from neural network medical semantic segmentation are often restricted to statistical summaries (Table 3), and *potentially* “cherry‐picked” clinical examples (Figure 3a). If reference datasets are accessible to authors for the given segmentation task, such as MICCAI^41^ challenge datasets, a statistical comparison between the proposed model and other published models may be provided to place the presented research in context with similar model results published elsewhere. Figure 3b shows the limitation of information being presented in this way where, despite excellent statistical metrics, the desired outcome quality (segmentation of bone and liver) was not achieved.

**TABLE 3:** Results of a single validation run of the same model used in Figure 5.

Metric	Calculation method	Training	Validation
Accuracy	Including background	97.0%	96.8%
Jaccard index	Including background	87.6%	87.6%
	Excluding background	83.2%	83.2%
DSC	Including background	93.2%	93.2%
	Excluding background	90.8%	90.8%
Recall	Including background	91.6%	91.6%
	Excluding background	88.0%	88.0%
Precision	Including background	95.1%	95.1%
	Excluding background	93.9%	93.9%

*Note*: Results were selected deliberately to show high descriptive statistic values, contrasting with the appearance of slices segmented using the same model. Patient 9 was used for validation results. Model details: Learning rate – 0.005, Dropout – 0.3, Optimizer – Adam, Loss Function – ComboLoss, CCE/DSC Weight – 5%/95%, Augmentation – False.

**FIGURE 4:**
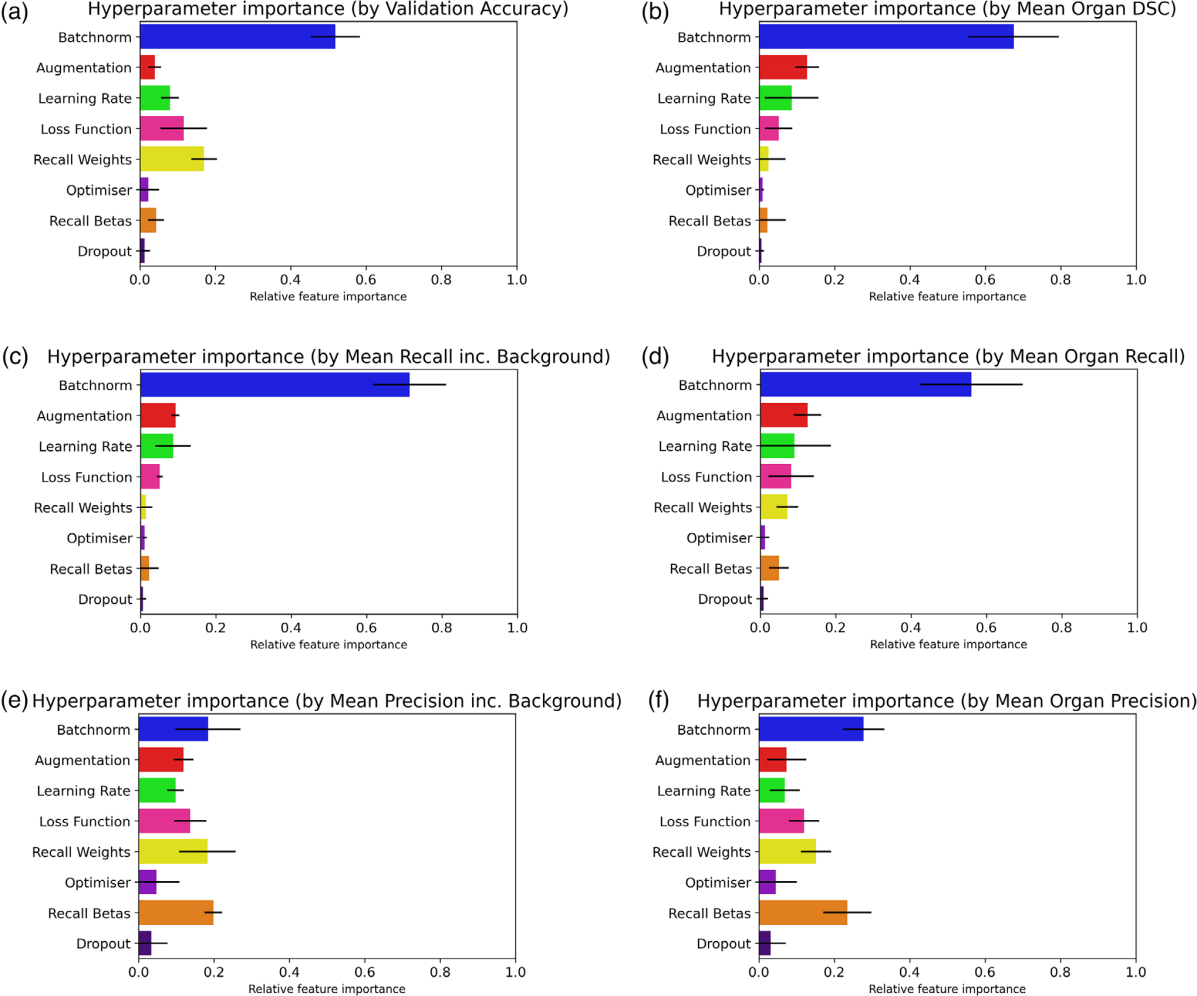
Comparison of relative hyperparameter importance using random forest regression analysis. “Importance” determined by Validation Accuracy and DSC with and without background (a‐b), Recall with and without background (c‐d), and Precision with and without background (e‐f). (95% error bars shown).

From Figure 4, random forest regression found batch normalization to be of greatest importance when model performance was assessed by Jaccard Index, DSC, and Recall. Interestingly, the hyperparameters that were most important to performance, as assessed by model precision, were found to change depending on whether segmentation of background was considered by the metric calculation. The use of precision and recall when results are calculated either with or without background can suggest that the model is working well despite it failing to achieve high performance on the clinical task. Understanding both their definition and clinical significance is important. Defined as the proportion of true positives as a fraction of total declared positives, precision is a metric that is like specificity and in the context of this work, a high precision score indicates that an identified pixel will likely belong to the identified class (organ). High precision may result from a conservative model which classifies only when certain, missing large amounts of that organ. When averaged over all organs this will be revealed, as the cases missed will have incorrectly been assigned to the background segment. If the background is excluded, the penalty is removed and a conservative, high‐precision model may be produced. Given medical images often contain a high proportion of “background”, and the consequences for missing something can be as severe as incorrect identification, the primary objective of medical segmentation models is as complete and correct as possible identification of organs, not identification of background. Batch normalization is relatively ubiquitous in model design, as it can improve training convergence and reduce training times, but for the case study used here batch normalization was found to be most influential on the results obtained. Conversely, the choice of optimizer was found to have little impact on model performance, despite many options for optimizers being available in the literature.

**FIGURE 5:**
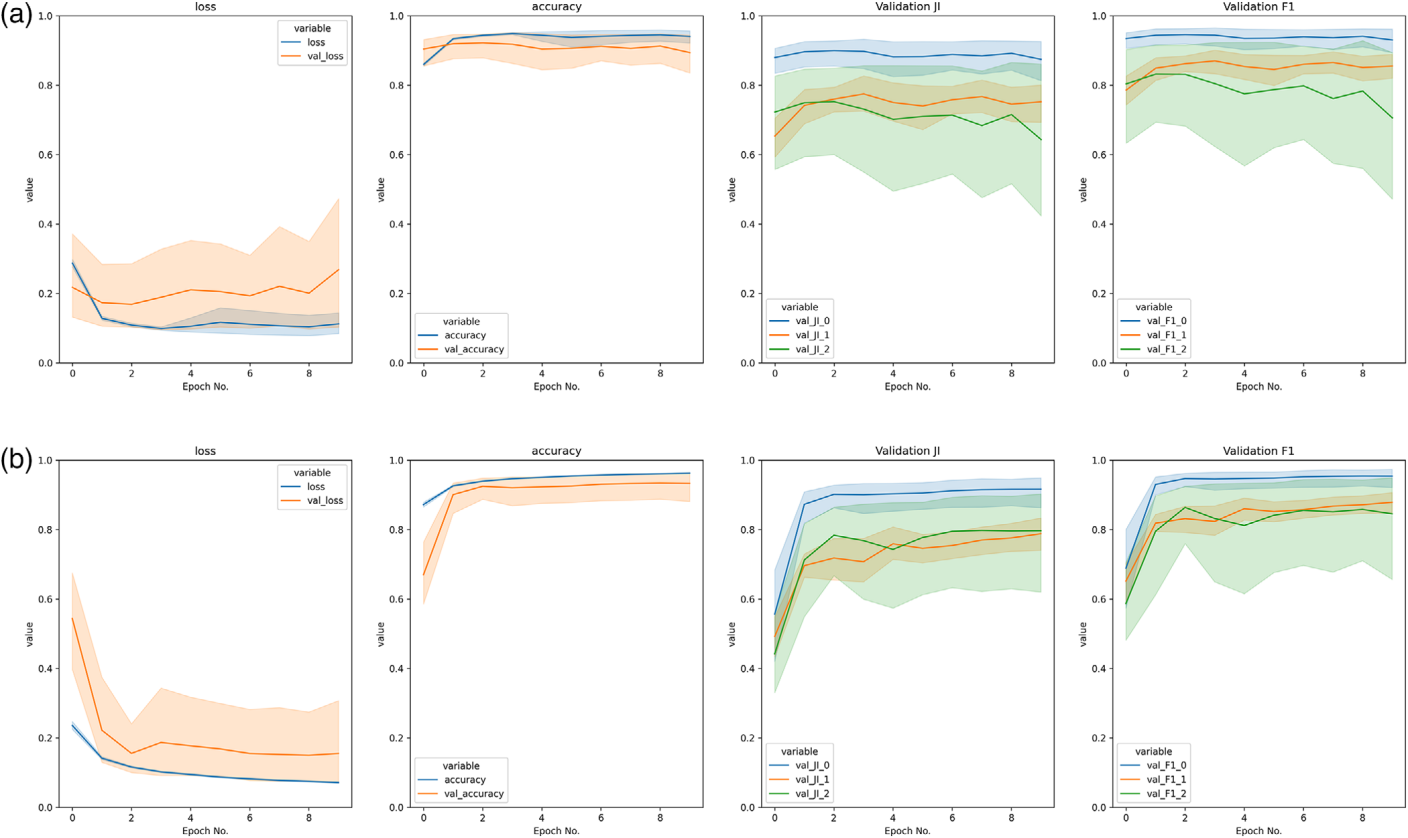
Examples of validation results from divergent (top) and convergent (bottom) training for the same model, without (top) and with (bottom) batch normalization (shaded region indicates 95% CI). Background, bone, and liver “_0”, “_1” and “_2”, respectively. For model parameters used, refer to caption of Table 3.

Figure 5 shows the loss, accuracy, Jaccard Index, and DSC from the validation patient series across cross‐validation folds. The two sub‐figures were obtained from two separate training runs, using hyperparameter combinations that differed only in the application of Batch Normalization. For this case study the impact of Batch Normalization was identified by most metrics as being the most impactful on overall model performance, which can be read from comparing the validation results over time in the top and bottom of Figure 5. The absence of Batch Normalization in this case led to a model that did not train (as shown by “val_loss” increasing rather than decreasing).

**FIGURE 6:**
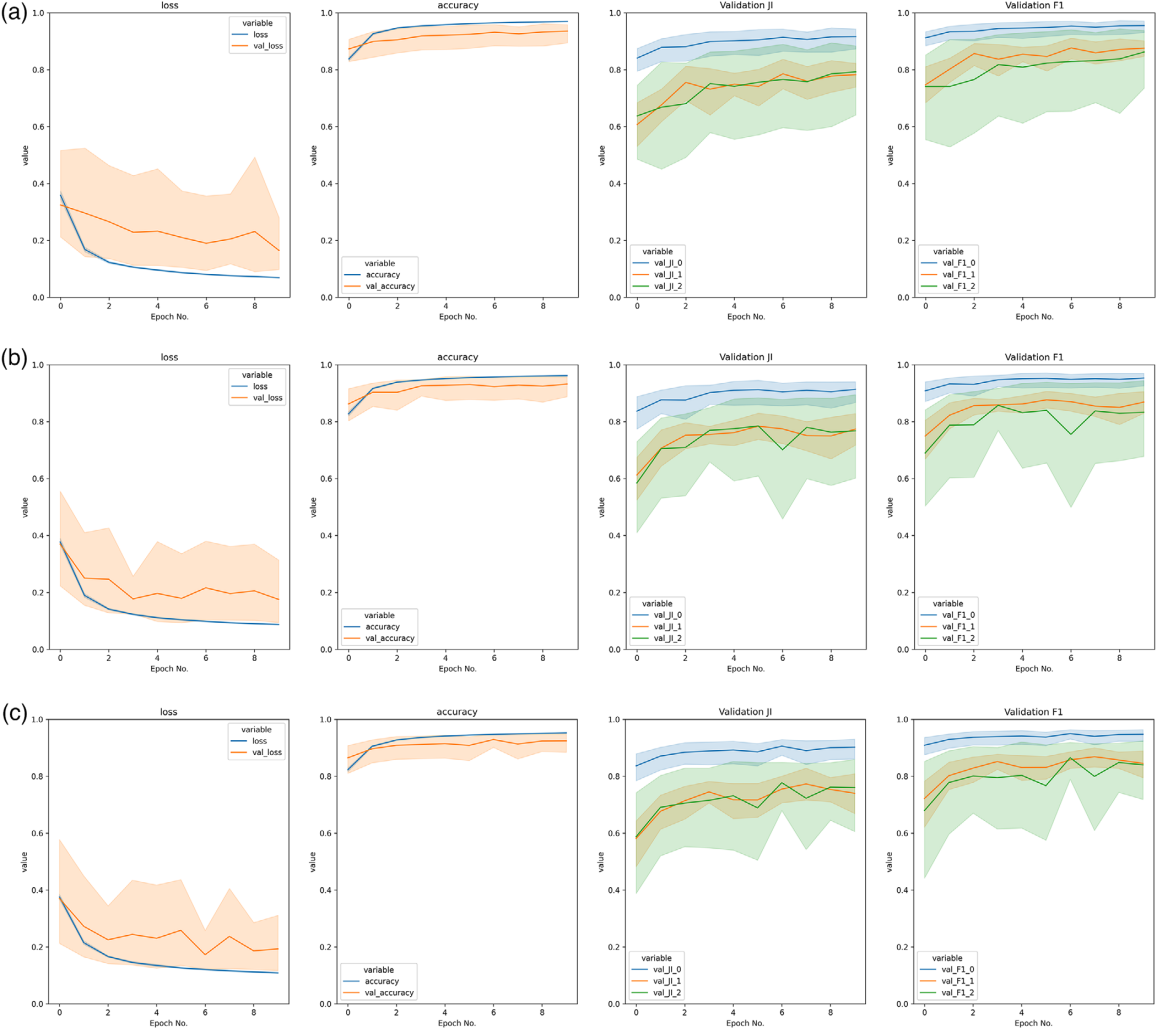
Examples of three models differing only in dropout selection. Dropout: (a) 0.1, (b) 0.3, (c) 0.5. Model details: Learning rate – 0.001, Dropout – variable, Optimizer – RMSProp, Loss function – ComboLoss, CCE/DSC Weight – 20%/80%, Augmentation – False, Batch Normalization – False.

Selection of dropout (0.1, 0.3, or 0.5), and choice of Optimizer (RMS propagation or Adam), unsurprisingly had the least impact on model training, with Figure 6 showing training diagrams varying only in dropout, with near identical outcomes. The limited impact of dropout is due to its’ role as a regularization parameter on neural network function, focused on preventing over‐fitting when unseen “validation” data are introduced.^42^ This was out of scope of this work due to the need to use unrestricted, publicly available data, supply of which is limited. The optimizers selected for use here are mathematically similar in formulation,^43^ with Adam having an added momentum term. Their similar performance has been seen in other works on comparative segmentation performance,^44^ but were used here as they were the best performing of the options investigated.

Visual investigation of training can provide information about hyperparameters, as well as from differences in metric values. Other training runs with nearly matched hyperparameters did not differ so strikingly, but when visually compared side by side, using either validation metric graphs or clinical segmentations, differences became clear. Although Table 3 shows excellent metric values, the model performance on each class is not equal. Models generally performed better when the class contained more pixels. In background‐heavy segmentation tasks, such as those typical to medicine, hyperparameter tuning for excellent background segmentation is likely to give the appearance of good statistics but not be clinically useful. The shaded regions in Figure 5 show a bootstrapped 95% confidence interval of performance as a function of epochs, calculated this way due to limited data available (one sample per fold). The trajectory of validation loss in Figure 5a indicates a problem in model development and reinforces the need to systematically test hyperparameter selections throughout model development.

## 4 DISCUSSION

Even though hyperparameter importance values identified here are true only for the dataset and model used in this case study, the methodology can be used in general to guide the development of small‐scale machine learning tools. It may be possible to guide model development by changing hyperparameters to improve performance metrics output during validation. However, given the potential biases introduced by hyperparameter selection and the consequences of this bias on clinical outcomes, more robust analysis such as a systematic grid search and/or random forest analysis of metrics, is likely to be beneficial. The clinical interpretation of performance metric values obtained by machine learning semantic segmentation should also be questioned, so model development decision‐making is informed by the most relevant information. Those newly entering the field of machine learning semantic segmentation are directed to an excellent summary of metric formalism and application in the literature.^45^ DSC in particular has been previously identified as misrepresentative of model performance,^46^ and general concerns have been raised around the reproducibility of model results.^47^

The model application described here utilized a publicly available dataset, yielding validation accuracy and validation DSC of 96.8% and 93.2%, respectively, in Table 3, which then presents what appears to be an acceptable level of model performance when compared in isolation to the DSC values around 75% that are typically seen in similar published works.^29^ This representation (or potential “misrepresentation”) is supported by Figure 3a, in which visual investigation does not immediately identify the differences between automated and human segmentation outcomes. Although this image has been deliberately selected to show maximum visual similarity, a deliberate selection of minimal visual similarity in Figure 3b shows a failure of the model, that does not identify much of the liver and ignores most of the ribs present in the image.

The use of random forest regression to determine relative hyperparameter importance allows the very large volume of results to be condensed for comparison and analysis. Figure 4e,f shows the large change in the importance of validation precision, after the removal of background. Given the propensity for medical images to contain relatively few pixels of interest amongst a sea of non interest pixel classes, a model that precisely segments by classifying most pixels as background pixels is of no use. With guidance from correctly chosen performance metrics, random forest regression is a relatively simple method for users to do a “before and after” of model performance after changes, in pursuit of optimization.

Understanding the use case and limitations of a selected hyperparameter combination and model design is important knowledge best obtained through experience. In tasks like the case study provided here, the most appropriate metrics will vary with the structures within an image being segmented. Common semantic segmentation tasks for medical applications often require the identification of early cancers, small lesions in early development, such as in demyelinating disorders or COVID, or segmentation of vascular pathology and histopathology, both of which are small by nature. In these cases, the most appropriate metrics would consider distance characteristics of the segmentations, such as the Hausdorff Distance and Mahalanobis Distance,^45^ rather than metrics which consider only the spatial characteristics, used in the example here for segmentation of whole organs.

Medical physics as a profession lends itself to the development and implementation of clinical machine learning tools. Although many possess or acquire some level of coding proficiency, several experiential pitfalls may beset a medical physicist when first commencing deep learning tool development. As MP3.0 outlines,^43^ the development of these tools is well aligned with good practice as it requires: applied scientific expertise, collaboration, the disciplinary extension of medical physics to include data science, and active involvement in clinical practice. However, when engaging in these projects, medical physicists need to bring their scientific rigor to maximize their overall contribution to the project's success. Those newly entering the machine learning arena may be concerned whether they have made the right choices and followed the right processes to obtain a good outcome. Systematic investigation of model hyperparameter importance should be used to assuage these issues. Soundly conducting such investigations is especially important in situations where powerful DL models are created from a relatively small set of data, as the potential for overfitting to the training data is ever present in such scenarios.

Six lessons learned during this work, which may be relevant for others involved in small‐scale model development, are included below:

### 4.1 General AI lessons

1. Do not accept a single summary performance metric value and/or a single good image result at face value.
2. Extreme caution is needed when transposing published “in‐house” models to your own clinic, especially if the original dataset cannot be provided due to patient confidentiality and restrictions from ethical approvals.
3. Programmers should be careful to not hand‐pick hyperparameters, either inadvertently via iterative training and evaluation, or relying entirely on previous experience to pick a single hyperparameter combination for the entire training process.

### 4.2 Small‐scale AI lessons

1. During model development, within the limits of available hardware, perform training with multiple combinations of hyperparameters. Although conventions exist for setting values, and may be a good guide for starting values, your dataset and model architecture may differ in ways not immediately apparent.
2. Training requires explicit and implicit selection of dozens of hyperparameters – test these regularly throughout development. Being able to determine which hyperparameters are the most important for your results will help improve your current performance and the stability of the model.
3. Presenting more information on model performance, with different hyperparameter settings improves transparency. This should be encouraged to prevent excess hyperparameter tuning and minimize the risk of overfitting.

### 4.3 Limitations

As mentioned earlier, the specifics of this study represent only the results obtained with the dataset and model used, however, there are several other important limitations of this work that must be acknowledged.

Model developers cannot have a priori knowledge of key hyperparameters for their research for all datasets, objectives, performance metrics, and model types. Optimization and development of models will require some manual selection of hyperparameters in almost all cases. The selection of a starting set of hyperparameters is decided by previous experience and information available in literature, balanced against time available for optimization.

Although comprehensive optimization of hyperparameters is possible,^15^, ^17^, ^48^ fully automating this process requires computational capacity and data access beyond that of many institutions. A grid search approach such as described here is relatively easy to implement and, although not the most computationally efficient, it can be immediately used to analyze the impact of development decisions.

Although high dimensionality can lead to a combinatorial explosion of hyperparameter configurations and potential computational wastage, these issues can be mitigated by narrowing grid sizes. Random searching has been shown to improve on grid searching,^49^ although neither option is guaranteed to find the optimal solution. Overall, grid search and random search are often good search strategies that can be readily employed; with other methods being more technically sophisticated and more difficult to deploy.

## 5 CONCLUSION

Practical limitations (time, money, and computational resources) may limit the scope for medical physicists to optimize the hyperparameters used in small‐scale machine learning models that they are likely to develop. Although it may be infeasible to find the truly optimal model configuration in all cases, simple sensitivity investigations and Random Forest regression analysis provide a basic assessment of reliability and validity, relevant to the dataset and application being investigated. Random forest regression analysis of large result datasets has also been found to be useful for condensing large metric outputs into readily interpretable figures showing relative hyperparameter importance, which can be useful for machine learning developers.

Given our recognized skill sets, medical physicists should expect to be involved in machine learning projects at various points throughout their careers. There is an obligation that we understand how to develop models cautiously and methodically. Sensitivity analysis can help add confidence to model validity, minimizing risks to clinical decision‐making and overall patient care.

## AUTHOR CONTRIBUTIONS

*Concept and design*: Chris Boyd and Eva Bezak. *Acquisition, analysis, or interpretation of data*: Chris Boyd, Wolfgang Mayer, and Mark Jenkinson. *Drafting of the manuscript*: Chris Boyd. *Critical revision of the manuscript for important intellectual content*: Timothy J. Kleinig, Joseph Dawson, and Gregory C. Brown. *Statistical analysis*: Chris Boyd and Eva Bezak. *Supervision*: Eva Bezak.

## CONFLICT OF INTEREST STATEMENT

The authors have no relevant competing interests to disclose.

## ETHICS STATEMENT

No human or animal participants were used in this work.

## Supporting information



Supporting Information
