# Supplementary material for: Hyperparameter selection for dataset‐constrained semantic segmentation: Practical machine learning optimization
Source: J Appl Clin Med Phys. 2024 Oct 10;25(12):e14542. doi: 10.1002/acm2.14542 (PMC11633816; doi:10.1002/acm2.14542)
Supplement: Supplementary file 1 — Supporting Information [file ACM2-25-e14542-s001.docx]

# Appendices

## Summary of statistical tools

Table 4: Summary of formulas for common model hyperparameters

| Parameter Type | Parameter Name | Formalism |
| --- | --- | --- |
| Optimizer Functions^8,31,48^ | Stochastic Gradient Descent (SGD) | $\theta_{t+1}=\theta_{t}- \eta_{t}\cdot\nabla J(\theta_{t})$ |
|  | Adam | $\theta_{t+1}= \theta_{t}-\frac{\eta}{\sqrt{\hat{\nu}_{t}}+\epsilon}\hat{m}_{t}where \hat{m}_{t}=\frac{m_{t}}{1-\beta_{1}^{t}} \& \hat{\nu}_{t}= \frac{\nu_{t}}{1-\beta_{2}^{t}}$ |
|  | Adagrad | $\theta_{t+1}= \theta_{t}-\frac{\eta}{\sqrt{G_{t}}+\epsilon}*\nabla J(\theta_{t})$ |
|  | Root Mean Square Propagation (RMSprop) | $\theta_{t+1}= \theta_{t}-[\gamma m_{t}+\frac{\eta}{\sqrt{\nu_{t+1}}+\epsilon}*\nabla J\left( \theta_{t} \right)]$ |
| Evaluation Metric | Recall | $Recall= \frac{True positive}{True positive+False Negative}$ |
|  | Precision | $Precision= \frac{True positive}{True positive+False Positive}$ |
|  | Accuracy | $Accuracy= \frac{True positive+True Negative}{True positive+False Positive+True Negative+False Negative}$ |
|  | Dice Similarity Coefficient (DSC) | $DSC= \frac{2\sum_{c=1}^{C} \sum_{i=1}^{N} g_{i}^{c}s_{i}^{c}}{\sum_{c=1}^{C} \sum_{i=1}^{N} g_{i}^{c}+ \sum_{c=1}^{C} \sum_{i=1}^{N} s_{i}^{c}}$ |
|  | Jaccard Index | $Jaccard Index= \frac{\sum_{c=1}^{C} \sum_{i=1}^{N} g_{i}^{c}s_{i}^{c}}{\sum_{c=1}^{C} \sum_{i=1}^{N} {(g}_{i}^{c}+s_{i}^{c}-g_{i}^{c}s_{i}^{c})}$ |
| Loss Function  ^33-36,49^ | Recall Favored (RF)^]^ | $L_{RF}=\frac{\sum_{c=1}^{C} \sum_{i=1}^{N} g_{i}^{c}s_{i}^{c}}{\sum_{c=1}^{C} \sum_{i=1}^{N} g_{i}^{c}s_{i}^{c}+\frac{\beta^{2}}{(1+\beta^{2})}\sum_{c=1}^{C} \sum_{i=1}^{N} ({1-g}_{i}^{c})s_{i}^{c}+\frac{1}{(1+\beta^{2})}\sum_{c=1}^{C} \sum_{i=1}^{N} g_{i}^{c}(1-s_{i}^{c})}$ |
|  | Dice Loss | $L_{Dice}= 1-\frac{2\sum_{c=1}^{C} \sum_{i=1}^{N} g_{i}^{c}s_{i}^{c}}{\sum_{c=1}^{C} \sum_{i=1}^{N} g_{i}^{c}+ \sum_{c=1}^{C} \sum_{i=1}^{N} s_{i}^{c}}$ |
|  | Tversky Loss | $L_{Tversky}= 1-\frac{\sum_{c=1}^{C} \sum_{i=1}^{N} g_{i}^{c}s_{i}^{c}}{\sum_{c=1}^{C} \sum_{i=1}^{N} g_{i}^{c}s_{i}^{c}+\alpha\sum_{c=1}^{C} \sum_{i=1}^{N} ({1-g}_{i}^{c})s_{i}^{c}+\beta\sum_{c=1}^{C} \sum_{i=1}^{N} g_{i}^{c}(1-s_{i}^{c})}$ |
|  | Focal Tversky Loss | $L_{Focal Tversky}= {(L}_{Tversky})^{\frac{1}{\gamma}}$ |
|  | Categorical Cross Entropy (CCE) | $L_{CCE}=-\frac{1}{N}\sum_{c=1}^{C} \sum_{i=1}^{N} {w_{c}g}_{i}^{c}s_{i}^{c}$ |
|  | Combo Loss | $L_{Combo}=w.L_{CCE}+\left( 1-w \right).L_{Dice}$ |
|  | Jaccard Distance | $L_{Jaccard}= 1-\frac{\sum_{c=1}^{C} \sum_{i=1}^{N} g_{i}^{c}s_{i}^{c}}{\sum_{c=1}^{C} \sum_{i=1}^{N} {(g}_{i}^{c}+s_{i}^{c}-g_{i}^{c}s_{i}^{c})}$ |
| Where: $\theta_{t+1}$ = Parameter at iteration *t+1*, $\eta_{t}$ = Learning rate (at iteration *t* where variable), $J$ = Loss function value, $m_{t}$ = Exponential moving average of the previous gradient $v_{t}$ = Square of the gradient, $\epsilon$ = Fixed non-negative small parameter. Prevents div0 errors, $G_{t}$ = Gradient at iteration *t,* $g_{i}^{c}$ = The true value of the i^th^ element of the c^th^ sample $s_{i}^{c}$ = The predicted value of the i^th^ element of the c^th^ sample, $\alpha, \beta, \gamma,w$ = Weighting factors, L_x_ = Type x loss, N = Total number of samples | | |
